# Hepatitis B virus X protein induces expression of alpha-fetoprotein and activates PI3K/mTOR signaling pathway in liver cells

**DOI:** 10.18632/oncotarget.2906

**Published:** 2015-01-21

**Authors:** Mingyue Zhu, Junli Guo, Wei Li, Yan Lu, Shigan Fu, Xieju Xie, Hua Xia, Xu Dong, Yi Chen, Ming Quan, Shaojiang Zheng, Keping Xie, Mengsen Li

**Affiliations:** ^1^ Hainan Provincial Key Laboratory of Carcinogenesis and Intervention, Hainan Medical College, Haikou, Hainan 571199, P. R. China; ^2^ Key Laboratory of Molecular Biology, Hainan Medical College, Haikou, Hainan 571199, P. R. China; ^3^ Department of Gastroenterology, Hepatology & Nutrition, The University of Texas MD Anderson Cancer Center, Houston, TX 77030, USA; ^4^ Department of Physiology and Pathophysiology, Hainan Medical College, Haikou, Hainan 571199, P. R. China; ^5^ Tumor Institute, Affiliated Hospital of Hainan Medical College, Haikou, Hainan 570102, P. R. China

**Keywords:** AFP, HBx, PI3K/mTOR signaling, Liver cells, Hepatocarcinogenesis

## Abstract

The hepatitis B virus (HBV)-X protein (HBx) induces malignant transformation of liver cells, and elevated expression of alpha-fetoprotein (AFP) is a significant biomarker of hepatocarcinogenesis. However, the role of AFP in HBV-related hepatocarcinogenesis is unclear. In this study, we investigated the regulatory impact of AFP expression on HBx-mediated malignant transformation of human hepatocytes. We found that HBV induced the expression of AFP before that of oncogenes, *e.g*., Src, Ras and chemokine (C-X-C motif) receptor 4 (CXCR4), and AFP activated protein kinase B (AKT) and mammalian target of rapamycin (mTOR) in HBV-related HCC tissues and in human liver cells transfected with HBx. Cytoplasmic AFP interacted with and inhibited phosphatase and tensin homolog deleted on chromosome 10 (PTEN), activating the phosphatidylinositol 3-kinase (PI3K)/AKT signaling pathway and promoting mTOR-mediated stimulation of the transcription factor hypoxia inducible factor-1α (HIF-1α), and therefore led to the activation of the promoters of Src, CXCR4, and Ras genes. On the contrary, reduced expression of AFP by siRNA resulted in the repression of p-mTOR, pAKT, Src, CXCR4, and Ras in human malignant liver cells. Taken together, for the first time our study indicates that HBx-induced AFP expression critically promote malignant transformation in liver cells through the activation of PI3K/mTOR signaling.

## INTRODUCTION

Hepatocellular carcinoma (HCC) is the most common type of liver cancer and currently accounts for approximately 610,000 deaths annually worldwide [1]. Hepatitis B virus (HBV) infection is a major risk factor for HCC [2, 3]. However, HBV cannot directly drive the malignant transformation of liver cells [4, 5]. As a multifunctional transactivator protein [4], HBV x protein (HBx) plays a crucial role in HBV-associated HCC development [6–8]. HBx is able to form a complex with the p53 tumor suppressor and inhibit its DNA binding and transcriptional activity [9, 10], thus activating the phosphatidylinositol 3-kinase (PI3K)/protein kinase B (AKT) signaling pathway and accelerating the transcription of oncogenes [11]. HBx promotes the proliferation and transformation of hepatocytes by hyperactivating oncogenic signals-including mitogen-activated protein kinase, PI3K, and wingless-related integration site [12–14]. HBx also prevents the apoptosis of liver cells by inhibiting the activity of caspase-3 [15] via PI3K/AKT signaling. However, the precise mechanisms by which HBx promotes the signal transduction of many oncogenic pathways remain unclear.

Alpha-fetoprotein (AFP) is an important biomarker of HCC. It is a strong, independent predicator of long-term HCC risk in HBV patients [16]. Recently, functional studies have revealed that LIM-homeobox family-4 (LHX-4) suppresses hepatocarcinogenesis through reducing AFP expression [17], and we have found that AFP interacts with and inhibits the activity of phosphatase and tensin homolog deleted on chromosome 10 (PTEN), stimulating the transduction of the PI3K/AKT signaling pathway [18]. PI3K is recruited and activated during the intracellular signal transduction of many growth factor receptors and has been implicated in the signaling of survival factors [19, 20]. The PI3K/AKT pathway also has been implicated in the development of liver carcinogenesis [21]; its activation contributes to the upregulation of Ras, Src, chemokine (C-X-C motif) receptor 4 (CXCR4) [22–26], mammalian target of rapamycin (mTOR), and hypoxia inducible factor-1α (HIF-1α) proteins [27–29]. We have recently confirmed that AFP promotes cancer cell proliferation [30], enhances *Ras* expression [31], and activates PI3K/AKT signals to enhance *Src* expression [32]. These observations suggest that the expression of AFP might be crucial in the malignant transformation of liver cells.

HBx overcomes p53-mediated repression of AFP expression [9], contributing to the development of HCC [33]. It is also capable of activating the 5′-upstream sequence of the *AFP* gene [34]. We have found that HBx drives the expression of AFP to activate PI3K/AKT signal to stimulate the expression of oncogenes in normal liver cells [31]. However, the exact mechanisms by which AFP and HBx synergize to drive malignant transformation of liver cells remains to be determined.

In our current study, we sought to determine whether AFP was a pivotal intracellular factor in HBx-mediated hepatocarcinogenesis. We hypothesized that AFP mediates the activation of the PI3K/AKT/mTOR pathway and promotes the HBx-induced expression of Ras, Src, and CXCR4 in hepatocytes.

## RESULTS

### AFP and oncogene expression during the development of HBV-related HCC

**Figure 1:**
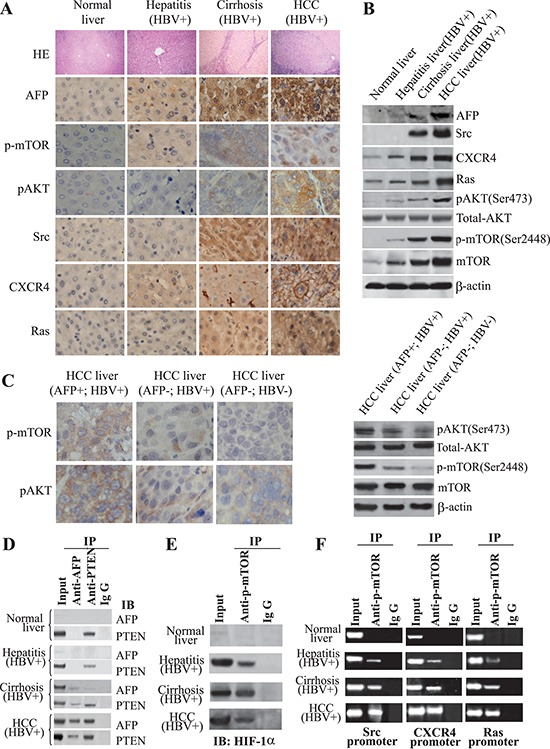
Influence of AFP on HBV-mediated malignant transformation of liver cells *in vivo* **(A)** Expression of AFP, p-mTOR(Ser2448), pAKT(Ser473), Src, CXCR4, and Ras was detected by immunohistochemistry (IH). IH images are shown at 400x and hematoxylin-eosin staining (HE) at 100x. **(B)** Expression of AFP, p-mTOR(Ser2448), pAKT(Ser473), Src, CXCR4, and Ras was detected by immunoblotting. **(C)** Expression and location of p-mTOR(Ser2448) and pAKT(Ser473) were assessed in AFP(+)/HBV(+), AFP(−)/HBV(+), and AFP(−)/HBV(−) HCC tissues by IH. pAKT(Ser473), total AKT, and p-mTOR(Ser2448) mTOR levels were detected by Western blotting. **(D)** The interaction of AFP and PTEN was assessed by Co-IP. **(E)** The interaction of p-mTOR(Ser2448) and HIF-1α was assessed by Co-IP. **(F)** p-mTOR(Ser2448) co-activated transcription of HIF-1α. Interaction of HIF-1α with the promoters of Src, CXCR4, and Ras was assessed by chromatin immunoprecipitation. Results are from one representative experiment of three. IgG – immunoglobulin G.

We studied the expression of AFP; downstream targets of the PI3K/AKT signaling pathway, AKT, pAKT (Ser473), and p-mTOR (Ser2448); and the oncogenes *Src*, *CXCR4*, and *Ras* in liver tissue samples from 63 patients by immunohistochemical staining and immunoblotting. AFP was expressed in all HBV-infected tissues, but at a much higher level in HCC tissues than in other liver tissues (Figure 1A and 1B). Ras, CXCR4, pAKT (Ser473), and p-mTOR (Ser2448) were expressed in all liver tissue samples, with progressive elevation of expression from normal liver tissue to HBV-infected tissue to cirrhotic tissue to HCC tissue. Src expression was limited to cirrhotic and HCC tissues (Figure 1A and 1B). Consistent with the results of Western blot analysis, the expression of AFP and CXCR4 mRNA were also increased as determined by quantitative RT-PCR analysis (Supplementary Figure 1A). The levels of pAKT (Ser473) and p-mTOR (Ser2448) were significantly higher in AFP+/HBV+ liver tissues than in AFP−/HBV+ or AFP−/HBV- liver tissues (Figure 1C). We confirmed the binding of AFP to PTEN in cirrhotic and HCC tissue samples (Figure 1D). We also observed interactions between HIF-1α and p-mTOR(Ser2448) in HBV-infected, cirrhotic, and HCC tissues (Figure 1E). Finally, to verify that p-mTOR(Ser2448) linked HIF-1α to trigger the transcription activity of downstream oncogenes, a ChIP assay was performed. The specific p-mTOR (Ser2448)-HIF-1α-DNA complex was immunoprecipitated using an anti-p-mTOR (Ser2448) antibody, and then to amplify the HIF-1α binding sequences of downstream genes promoter. As shown in Figure 1F, anti-p-mTOR (Ser2448) antibodies, but not control IgG, amplified the predicted size DNA fragments from the precipitates of the samples. These results indicated that p-mTOR(Ser2448)-enhanced HIF-1α binding to the promoters of *Src*, *CXCR4*, and *Ras* genes.

### Timing of AFP and oncogene expression during HBx-induced malignant transformation of human normal liver cells *in vitro*

**Figure 2:**
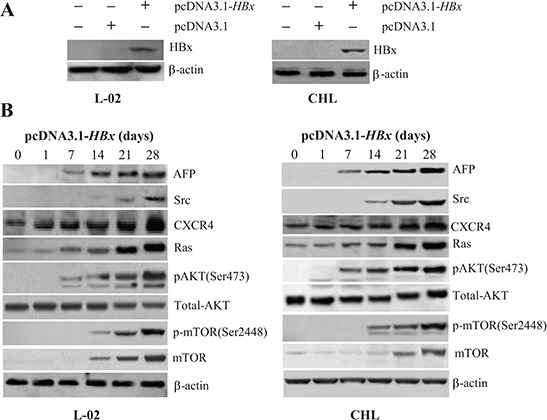
Effects of pcDNA3.1-*HBx* on the expression of AFP, Src, CXCR4, Ras, pAKT(Ser473), and p-mTOR(Ser2448) in liver cells **(A)** L-02 and CHL liver cells were transfected with pcDNA3.1-*HBx* and expression of HBx was measured 48 hours later by Western blotting. **(B)** Expression of Src, CXCR4, Ras, pAKT(Ser473), and p-mTOR(Ser2448) was measured by Western blotting 1, 7, 14, 21, and 28 days after transfection. Results are from one representative experiment of three.

We transfected a vector expressing the HBV protein HBx, pcDNA3.1-*HBx*, into human liver cell lines L-02 and CHL and measured the impact of pcDNA3.1-*HBx* on the expression of AFP and the oncogenes *Src*, *CXCR4*, and *Ras*. The pcDNA3.1-*HBx*-mediated induction of HBx expression in L-02 and CHL cells was evident 2 days after transfection and remained elevated between 7 and 28 days after transfection (Figure 2A). Expression of AFP was emerged after transfected with pcDNA3.1-*HBx* for 7 days and persisted increasing after 28 days. Expression of CXCR4 and Ras was enhanced 7 day after transfection and increased for 28 days in both L-02 and CHL cells (Figure 2B). The expressions of AFP and CXCR4 were further confirmed at the mRNA levels by quantitative RT-PCR analysis (Supplementary Figure 1B). Src expression was elevated 14 days after transfection in both L-02 and CHL cells (Figure 2B).

### Interaction of AFP and PTEN in malignant liver cells

**Figure 3:**
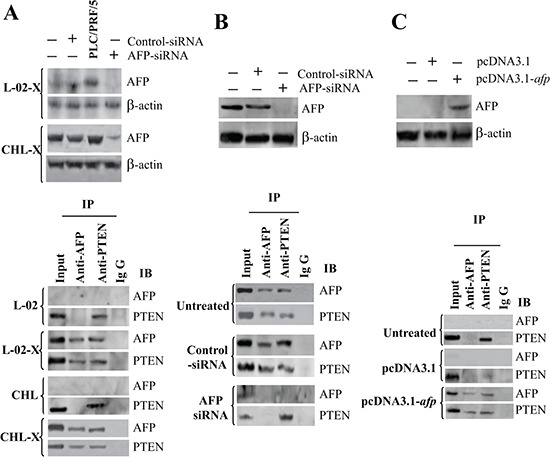
The interaction and co-localization of AFP with PTEN in liver cells **(A)** The pcDNA3.1-*HBx* and a control vector were transfected into L-02 and CHL cells, and the expression of AFP and the interaction of AFP with PTEN were determined by Western blotting and Co-IP. **(B)** The expression of AFP and interaction of AFP with PTEN were also determined by Western blotting and Co-IP in AFP-siRNA-transfected PLC/PRF/5 cells. **(C)** The expression of AFP and interaction of AFP with PTEN were also determined by Western blotting and Co-IP in pcDNA3.1-*afp*-transfected HLE cells.

PTEN is a critical inhibitor of PI3K activation [38, 39], and the PI3K/AKT signaling pathway plays a pivotal role in the development of HBV-related HCC [40]. To investigate the interaction between AFP and PTEN, we used L-02-X, and CHL-X, and PLC/PRF/5 cells. Immunoblotting analysis showed that *HBx* transfection induced AFP expression in L-02 and CHL cells, and AFP-siRNA significantly decreased the expression of AFP in those cells. Co-IP indicated that AFP bound with PTEN in L-02-X, CHL-X, and PLC/PRF/5 cells, but the binding vanished when those cells were transfected with AFP-siRNA (Figure 3A and 3B). AFP binding with PTEN was also observed by immunoblotting and Co-IP in non-AFP-producing HLE HCC cells. Twenty-four hours after transfection of the HLE cells with pcDNA3.1-*afp,* the expression of AFP was stimulated as measured by immunoblotting, and AFP binding with PTEN was evident as demonstrated by Co-IP (Figure 3C). We further applied laser confocal microscopy to observe the location of the AFP-PTEN complex in L-02-X, CHL-X, and PLC/PRF/5 cells. The results indicated that AFP and PTEN were superimposed in the cytoplasm (Supplementary Figure 2). Laser confocal microscope software analysis indicated contiguous distant adequate delivery of fluorescent energy between AFP and PTEN molecules (Supplementary Figure 3). Thus, our data clearly suggested a direct interaction between AFP and PTEN in malignant liver cells.

### Role of AFP in p-mTOR(Ser2448) translocation into the nucleus and HIF-1α-induced expression of Src, CXCR4, and Ras in malignant liver cells

**Figure 4:**
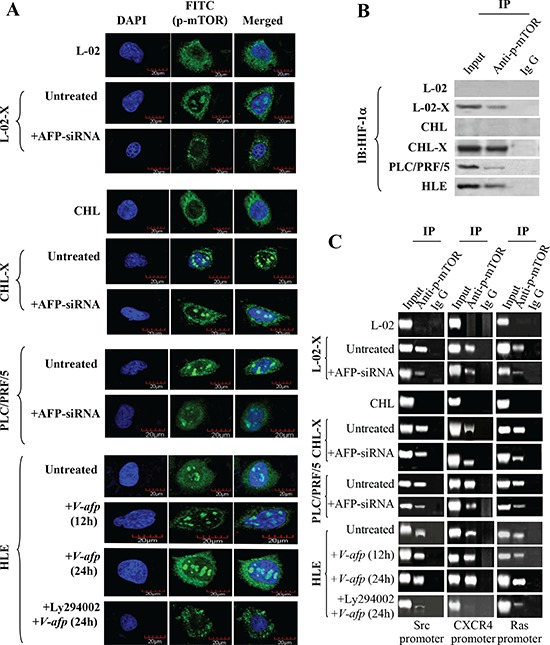
Activation of mTOR by AFP and upregulation of HIF-1α transcription and expression of Src, CXCR4, and Ras in liver cells **(A)** AFP induced p-mTOR(Ser2448) nuclear translocation in L-02, CHL, PLC/PRF/5, HLE, L-02-X, and CHL-X cells was observed by laser confocal microscopy. **(B)** Interaction of p-mTOR(Ser2448) with HIF-1α in L-02, CHL, PLC/PRF/5, HLE, L-02-X, and CHL-X cells was detected by Co-IP. **(C)** mTOR was activated by AFP to co-activate the transcription of HIF-1α, regulating the expression of Src, CXCR4, and Ras in L-02, CHL, PLC/PRF/5, HLE, L-02-X, and CHL-X cells and in pcDNA3.1-*afp*-transfected and Ly294002-treated HLE cells. The transcription coactivator of p-mTOR(Ser2448) in HIF-1α interaction with promoters of Src, Ras, and CXCR4 genes was analyzed by chromatin immunoprecipitation. Results are from one representative experiment of three.

The PI3K/AKT signaling pathway regulates gene expression through activation of mTOR. We explored the influence of AFP on the activity of mTOR. Laser confocal microscope observations revealed that in the normal human liver L-02 and CHL cell lines, p-mTOR(Ser2448) was located only in the cytoplasm, but p-mTOR(Ser2448) was located in the cytoplasm and nucleus of the AFP-producing and HBx-expressing malignant liver cell lines L-02-X, and CHL-X, and in HCC and PLC/PRF/5 cells. Transfection of AFP-siRNA into those cells repressed p-mTOR(Ser2448) migration to the nucleus, and p-mTOR(Ser2448) was activated and migrated into the nucleus when the non-AFP-producing/non-HBV-infected cell line HLE were transfected with an AFP-expression vector, pcDNA3.1-*af*p (V-afp), however, which can be obviously suppressed by administration of PI3K/Akt singling inhibitor Ly294002 (10 μM) (Figure 4A), suggesting that activation of PI3K/Akt pathway was crucial for AFP-mediated p-mTOR(Ser2448) migration into the nucleus. A previous study reported that p-mTOR(Ser2448) is a co-factor of transcription and synergizes with HIF-1α to regulate the expression of vascular endothelial growth factor in HCC cells [41]. Therefore, we investigated the interaction of p-mTOR(Ser2448) and HIF-1α and the co-localization of p-mTOR(Ser2448) and HIF-1α in normal human liver L-02, CHL cells, L-02-X, CHL-X, PLC/PRF/5, and HLE cells by laser confocal microscope (Supplementary Figure 4). Moreover, co-IP results also demonstrated that p-mTOR(Ser2448) bound with HIF-1α in L-02-X, CHL-X, PLC/PRF/5, and HLE cells but not in L-02 and CHL cells (Figure 4B). The p-mTOR(Ser2448) enhanced HIF-1α interaction with the promoter sequences of Src, CXCR4, and Ras in L-02-X, CHL-X, PLC/PRF/5, and HLE cells but not in L-02 and CHL cells. HIF-1α interactions with the Src, CXCR4, and Ras gene promoters were suppressed in AFP-siRNA-transfected L-02-X and CHL-X cells and PLC/PRF/5 cells. The binding effect was weak in the HLE cells but was enhanced when transfected with pcDNA3.1-*afp*, moreover, the increased interaction of HIF-1α with downstream genes promoter were dependent on the activation of PI3K/Akt pathway (Figure 4C) We concluded that AFP accelerated the migration of p-mTOR(Ser2448) into the nucleus and stimulated the interaction of HIF-1α with the promoter sequences of *Src*, *CXCR4*, and *Ras*.

### Role of AFP in PI3K/mTOR signaling pathway activation and expression of Src, CXCR4, and Ras protein expression

**Figure 5:**
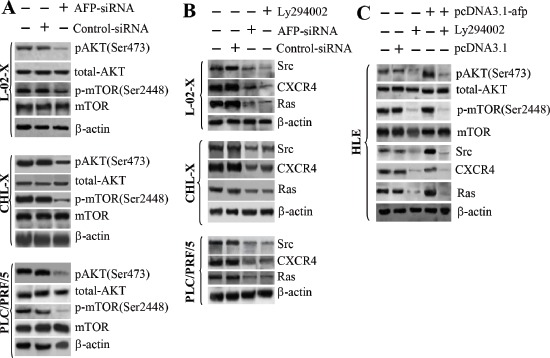
Effects of AFP on the expression of pAKT(Ser473), p-mTOR(Ser2448), Src, Ras, and CXCR4 in L-02, CHL, PLC/PRF/5, HLE, L-02-X, and CHL-X cells **(A)** The impact of AFP-siRNA on the expression of pAKT(Ser473) and p-mTOR(Ser2448) was assessed by immunoblotting. **(B)** The impact of the AFP-siRNA vector on the expression of Src, CXCR4, and Ras was detected by Western blotting. **(C)** The expression of pAKT(Ser473), p-mTOR(Ser2448), Src, CXCR4, and Ras in HLE cells was assessed by Western blotting after transfection with pcDNA3.1-*afp* for 48 hours. Results are from one representative experiment of three.

We further analyzed the effects of AFP on PI3K/AKT signaling pathway transduction in L-02-X, CHL-X, PLC/PRF/5, and HLE cells using the PI3K inhibitor Ly294002. Immunoblotting results indicated that AFP-siRNA, which is functionally similar to Ly294002, repressed the expression of pAKT(Ser473) and p-mTOR(Ser2448) (Figure 5A) and inhibited the expression of Src, CXCR4, and Ras (Figure 5B). The expression of pAKT(Ser473), p-mTOR(Ser2448), Src, CXCR4, and Ras was enhanced when HLE cells were transfected with pcDNA3.1-*afp*, and these effects were attenuated when the cells were treated with Ly294002 (Figure 5C). Thus, AFP represented a pivotal factor in the activation of the PI3K/mTOR signaling pathway.

## DISCUSSION

In the present study, we have provided several lines of evidence supporting AFP involvement in the early stages of HBV-related hepatocarcinogenesis. Specifically, we have found that expression of AFP occurs prior to upregulation of the oncogenes *Src* and *Ras*. In both cirrhotic and HCC liver tissues, in which AFP is significantly upregulated, Co-IP analyses revealed that AFP interacts with PTEN, stimulates pAKT(Ser473) and p-mTOR(Ser2448) expression, and enhances the binding of HIF-1α to the promoters of Src, Ras, and CXCR4. Engineered expression of HBx stimulated AFP expression before Src and Ras expression. Thus, HBx induces malignant transformation of liver cells via the upregulation of AFP, which promotes the proliferation of HCC cells by activating the PI3K/mTOR signaling pathway and suppressing PTEN activity.

Prior studies have shown that AFP is an important biomarker of HCC [16] and is upregulated in HBV-transfected human hepatoma cells [34, 42]. In our current study, we found that the expression of AFP was promoted when normal human liver cells were transfected with pcDNA3.1-HBx. Consistently, HBx activates the 5′-upstream sequence of the *AFP* gene [43]. The increased expression of AFP appears to occur before the expression of other oncogenes, *e.g*., *Src* and *Ras*. It is known that phosphorylation of AKT is enhanced by PI3K, activating a cascade of downstream targets, including p-mTOR(Ser2448), and accelerated the nuclear import of certain transcription factors. The synergistic effect of p-mTOR(Ser2448) with HIF-1α on the expression of oncogenes has also been previously reported [41, 44]. We found that AFP plays a role in enhancing the nuclear entry of p-mTOR(Ser2448). Interestingly, p-mTOR(Ser2448) interacted with HIF-1α in the cytoplasm, and HIF-1α bound to the promoters of Src, CXCR4, and Ras, strongly indicating that AFP promoted the nuclear import of p-mTOR(Ser2448) and HIF-1α.

Although HBx may trigger PI3K activity, reducing apoptosis of transformed liver cells [15], the molecular mechanisms of this process have not been well understood. AFP, an onco-embryo protein, is expressed at a high level during development but silenced for 2 years after birth [44, 45]. *AFP* gene expression is reactivated when liver cells are infected with HBV [9]. The mechanism underlying this change is unclear. In our current study, we found that the expression of AFP was triggered in normal human liver cells transfected with an HBx expression vector and that, in AFP-producing HCC cells, transfection with the AFP-siRNA vector repressed AFP expression and decreased the levels of pAKT(Ser473), p-mTOR(Ser2448), Src, CXCR4, and Ras. These effects were similar to those achieved by the use of Ly294002, a specific PI3K inhibitor. Moreover, when cells that do not produce AFP were transfected with the AFP expression vector, the expression of pAKT(Ser473) and p-mTOR(Ser2448) were increased, and this increased expression could be attenuated by Ly294002. Given that Ly294002 inhibits the phosphorylation of AKT by PI3K and that Ly294002 also restrains AFP-stimulated expression of pAKT(Ser473) and p-mTOR(Ser2448), HBx may initiate the activation of PI3K/mTOR at least in part through inducing AFP expression.

**Figure 6:**
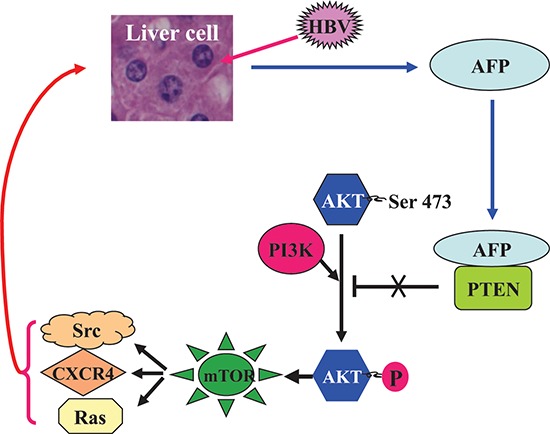
A schematic role of AFP in the HBV-driven malignant transformation of liver cells HBV infection stimulates the expression of AFP, which binds to PTEN and attenuates its function, thus activating the PI3K/AKT/mTOR pathway and promoting the HBx-induced expression of Ras, Src, and CXCR4 in hepatocytes, and collectively driving malignant transformation of liver cells.

Furthermore, we have found that AFP promoted nuclear translocalization of p-mTOR(Ser2448), resulting in HBx-stimulated expression of Src, CXCR4, and Ras in normal human liver cells, as outlined in the schematic diagram of HBV-induced malignant transformation of liver cells (Figure 6). Accumulating evidence suggests that HBx maintains the survival of HBV-infected liver cells by activating PI3K, Src, and Ras [46, 47]. Recently, investigation indicated that AFP activated PI3K/AKT signal pathway to promote proliferation of hepatoma cells [48]. In this study, our results demonstrated that HBx stimulates the neoplastic transformation of normal cells and tumor induction by AFP triggering of the PI3K/mTOR signal to promote expression of Src, Ras, and CXCR4, which are known to promote the progression, invasion, and metastasis of cancer cells [49]. Consistently, HBV-related HCC in patients with high levels of serum AFP appears to have a high metastatic potential [50]. Those studies suggest that AFP could be critically important at both the early and advanced stages of hepatocarcinogenesis.

In summary, HBV infection stimulates AFP expression, and AFP is a pivotal intracellular factor, activating the PI3K/AKT/mTOR pathway and promoting the HBx-induced expression of *Ras*, *Src*, and *CXCR4* in hepatocytes. Collectively, our results further substantiate the critical role of AFP and HBx in the early stage of hepatocarcinogenesis. Therefore, limiting AFP expression and function may represent a potential intervention strategy to attenuate HBV-related hepatocarcinogenesis.

## MATERIAL AND METHODS

### Clinical specimen collection

Archived clinical specimens were originally collected during hepatectomy of 63 patients at Hainan Provincial People's Hospital between 2008 and 2012. Of the 63 patients, 44 were male and 19 were female. The ages ranged between 22–76 years with an average age of 49 years (Supplementary Table 1). All enrolled patients were treated with radical surgery and received no other treatments. HBV infection was diagnosed by a serum hepatitis B surface antigen test, and circulating AFP plasma level was measured by enzyme-linked immunosorbent assay. Clinical data were obtained by retrospective chart review. Follow-up was available for all patients. A liver tissue about 2.0 × 2.0 × 2.0 cm was obtained from each patient immediately after the surgery. About 1.0 × 1.0 × 1.0 cm tissue samples were fixed in 10% formalin, embedded in paraffin, and routinely stained with hematoxylin and eosin. Specimens were assessed blindly and independently by two pathologists. In case of inter-observer disagreement, final decisions were achieved by general consensus. All selected patients were diagnosed by histopathologic evaluation. About 1.0 × 1.0 × 1.0 cm tissue specimens were stored in formalin and liquid nitrogen. The study protocol was approved by the Ethical Committee of Hainan Provincial Peoples' Hospital and the Science Investigation Ethical Committee of Hainan Medical College. Written informed consent was obtained from all participants.

### Immunohistochemical staining

The tumor samples of six patients were randomly selected for immunohistochemical staining. Following deparaffinization and antigen retrieval, the slides were blocked with 3% hydrogen peroxide for 10 minutes and then incubated with mouse anti-AFP (Cat.#sc-8399), goat anti-Src (Cat.#sc-6096), mouse anti-CXCR4 (Cat.# sc-53534), mouse anti-N-ras (Cat.#sc-31) (all from Santa Cruz Biotechnology), rabbit anti- phosphorylated AKT1 (pAKT; Ser473, Cat.#ab8932) and anti-phosphorylated mTOR (p-mTOR; Ser2448, Cat.#ab84400) (Abcam Biotech Company, Cambridge, UK) at 4°C overnight. After washing, sections were incubated with secondary goat anti-mouse antibodies (Merck-Calbiochem) at room temperature for 60 minutes and then developed with 3,3-diaminobenzidine chromogen solution in 3,3-diaminobenzidine buffer substrate (Merck Chemicals). Sections were visualized with 3,3-diaminobenzidine and counterstained with hematoxylin. All sections were visualized by microscope (Olympus).

### Cell lines

Human normal liver cell lines (L-02 and Chang liver [CHL]) were purchased from the Shanghai Institution of Cellular Biology, Science Academy of China and were cultured in RPMI 1640 medium supplemented with 10% fetal calf serum. The AFP-producing and HBV-infected cell line PLC/PRF/5 and the non-AFP-producing/non-HBV-infected cell line HLE were gifts from the Department of Cell Biology, Peking University and were maintained in Dulbecco's modified Eagle's medium supplemented with 10% fetal calf serum. All cell lines were cultured at 37°C in a humidified atmosphere containing 5% CO_2_.

### Generation of HBx- and AFP-expressing constructs

Construction of the HBx-expressing construct (pcDNA3.1-*HBx*) and the primer used for HBx gene amplification have been previously described [31]. The construct pcDNA3.1-*HBx* was transfected into L-02 and CHL cells to measure the impact of pcDNA3.1-*HBx* on the expression of AFP and the oncogenes *Src*, *CXCR4*, and *Ras*. Stably transfected L-02 and CHL cells were selected using G418 (Cat No. 30–234-CR, Mediatech Inc, Manassas, USA) and named L-02-X and CHL-X, respectively. Construction of the full-length AFP-expressing vector (pcDNA3.1-*afp*) and Full-length human AFP cDNA was obtained by RT-PCR amplification the primer used for amplification of the *afp* gene have been previously described [18].

### RNA interference assay and AFP-expressing vector construction

RNA interference was used to assess the effect of AFP on the PI3K/AKT signaling pathway as described previously [18, 35], and the AFP-expressing vector construct has been described previously [36].

### RNA extraction and quantitative RT-PCR

Total RNA was extracted with Trizol reagent (Invitrogen, Carlsbad, CA, USA). One μg of total RNA was used as template to generate the cDNA by oligo (dT18) using Fermentas RT System (cat.#K1622, Thermo Scientific, Guangzhou, China). The pairs of primers were synthesized by Sangon Biotech Co., Ltd. (Shanghai, China) as following, *AFP*: forward 5′-ccaacaggaggccatgctt-3′, *AFP* reverse 5′-gaatgcaggagggacatatgttt-3′; *CXCR4* forward 5′-atcagtctggaccgctacct-3′, *CXCR4* reverse 5′-ccaccttttcagccaacagc-3′; *β-actin* forward 5′-tgacgtgg acatccgcaaag-3′, *β-actin* reverse 5′-ctggaaggtggacag cgagg-3′. PCR was conducted using the LightCycler480 II instrument [Roche (China) Ltd., Guangzhou, China]. The total volume was 20 μl, which include 10 μl SYBR Green I PCR Master Mix (TOYOBO, OSAKA, Japan), 0.4 μl forward primer (10 μM), 0.4 μl reverse primer (10 μM), 2 μl cDNA and 7.2 μl ddH_2_O. The PCR amplification was as follow: after denaturation at 95°C for 1 min, 45 PCR cycles were performed including 95°C for 15 sec, 60°C for 60 sec. The relative abundance of target mRNAs were determined from the CT values and plotted as the fold change compared with that of the control groups.

### Western blotting and co-immunoprecipitation (Co-IP) analysis

Western blotting was employed to assess the protein levels of AFP, pAKT (Ser473), p-mTOR (Ser2448), Src, CXCR4, and Ras. Co-IP was employed to assess the binding of AFP to PTEN, p-mTOR(Ser2448), and HIF-1α in patient specimens and cell lines as described previously [18, 36].

### Fluorescence resonance energy transfer (FRET) analysis of intracellular co-localization of AFP and PTEN

Fluorescence resonance energy transfer was employed to investigate the interaction between AFP and PTEN in L-02, L-02-X, CHL, CHL-X, PLC/PRF/5, and HLE cell lines as previously described [36]. Briefly, cells were fixed in paraformaldehyde (4%), and rabbit anti-PTEN antibody and mouse anti-human AFP antibody (Santa Cruz Biotechnology) were added and incubated for 12 hours. Secondary goat anti-mouse or anti-rabbit immunoglobulin G conjugated with fluorescence isothiocyanate (FITC) or tetramethylrhodamine isothiocyanate (Zhongshan Biol Tech Co., Beijing) was applied for 2 hours, followed by the addition of 100 μL of 4′,6-diamidino-2-phenylindole (DAPI) (1 μg/mL). Cells were visualized with the Leica TCS-NT SP2 laser confocal microscope and associated software (Leica Camera) and analyzed by fluorescence resonance energy transfer.

### Laser confocal microscopy observation

The staining procedure for confocal microscopy has been previously described [35]. Briefly, cells were fixed in 4% paraformaldehyde and incubated with rabbit anti-human p-mTOR (Ser2448) antibody for 12 hours. FITC-conjugated secondary anti-rabbit immunoglobulin G was added and incubated for 2 hours, followed by the addition of 100 μL DAPI (1 μg/mL) and 30 minutes of incubation. Cells were visualized with the Leica TCS-NT SP2 laser confocal microscope (Leica Camera).

### Chromatin immunoprecipitation assay

The interaction of p-mTOR (Ser2448) with the promoter sequences of *Src*, *CXCR4*, and *Ras* genes in liver tissues and cell lines was assessed via chromatin immunoprecipitationassay, as previously described [18, 37]. Immunoprecipitation with p-mTOR (Ser2448) antibody and polymerase chain reaction was used to analyze HIF-1α binding with the promoter of aim genes. The PCR primers used were: *Src*: Forward, 5′-ctctctgtcatcccagttctcg-3′, Reverse, 5′-aggtgccacagccagtcaa-3′; *CXCR4*: Forward, 5′-ggcagcaggtagcaaagtga-3′, Reverse, 5′-agacaatgtaactc gctccaaga-3′; *Ras*: Forward, 5′-gctccgggtcagaattggc-3′, and Reverse, 5′-accgcccattcctcactcc-3′.

## SUPPLEMENTARY FIGUREs AND TABLE



## Abbreviations

AFP: alpha-fetoprotein
Co-IP: co-immunoprecipitation
PI3K: phosphatidylinositol 3-kinase
AKT: protein kinase B
pAKT: phosphorylated AKT (Ser473)
mTOR: Mammalian target of rapamycin
p-mTOR: phosphorylated mTOR (Ser2448)
PTEN: phosphatase and tensin homolog deleted on chromosome 10
HIF-1α: hypoxia inducible factor-1α
