# Supplementary material for: Hepatitis B virus X protein induces expression of alpha-fetoprotein and activates PI3K/mTOR signaling pathway in liver cells
Source: Oncotarget. 2015 Jan 21;6(14):12196–208. doi: 10.18632/oncotarget.2906 (PMC4494932; doi:10.18632/oncotarget.2906)
Supplement: Supplementary file 1 [file oncotarget-06-12196-s001.pdf]

SUPPLEMENTARY FIGURES AND TABLE

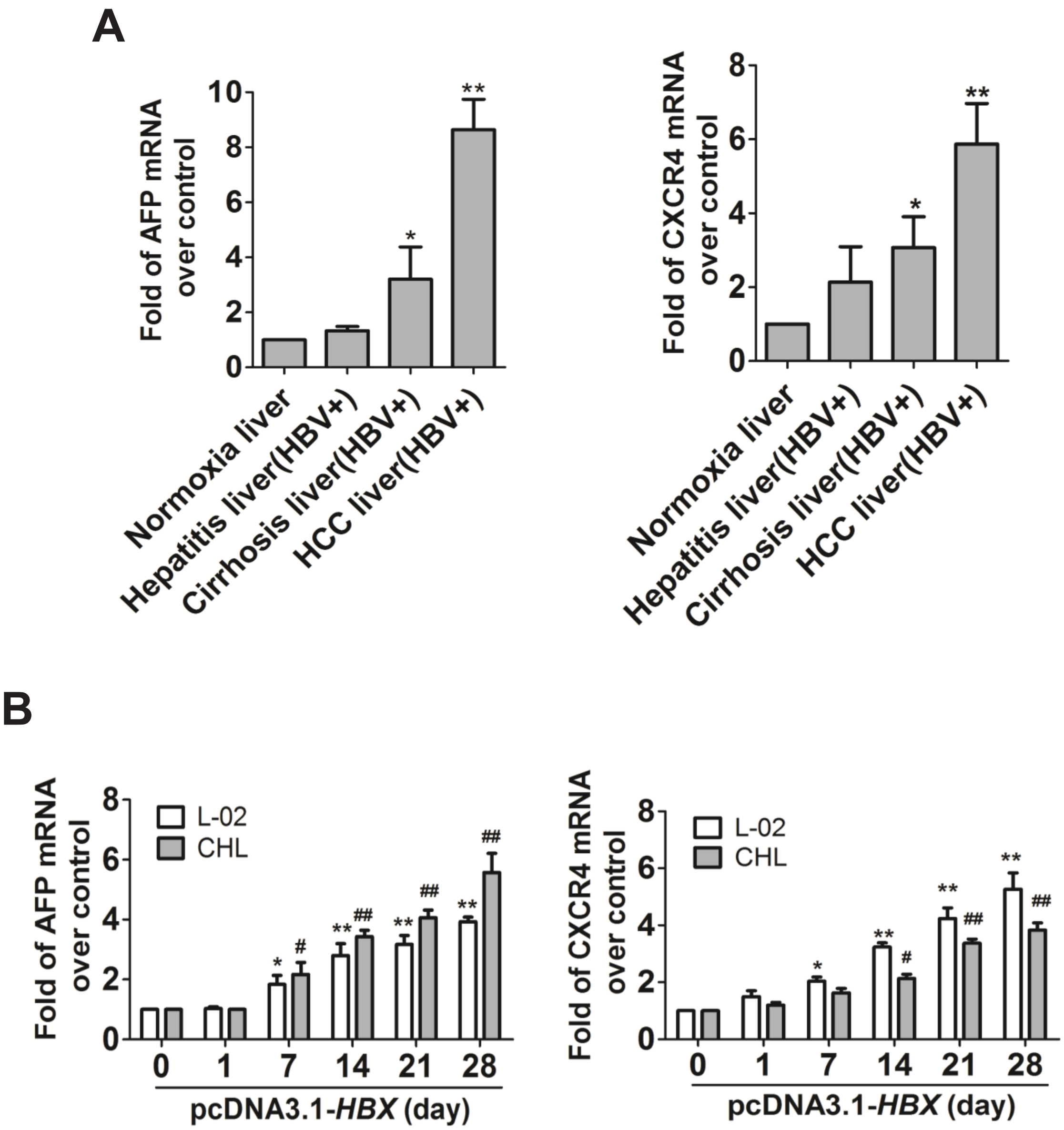

Supplementary Figure S1: Effects of HBx in expression of AFP or CXCR4 mRNA in clinical patients and human hepatoma cells. **(A)** Total RNA in patients' tissue samples were extracted, AFP or CXCR4 mRNA was detected by quantitative RT-PCR, the relative abundance of target mRNAs were determined from the CT values and plotted as the fold change compared with that of the control groups, **P* < 0.05, ***P* < 0.01 vs normal liver and hepatitis liver groups. **(B)** L-02 cells or CHL cells were transfected with pcDNA3.1-*HBx* vectors for 0, 7, 14, 21 and 28 days, total RNA in the cells were extracted, AFP or CXCR4 mRNA was detected by quantitative RT-PCR, the relative abundance of target mRNAs were determined from the CT values and plotted as the fold change compared with that of the control groups, *#*P* < 0.05, **##*P* < 0.01 vs day 0 and day 1 groups. One of three independent experiments with similar results was represented.

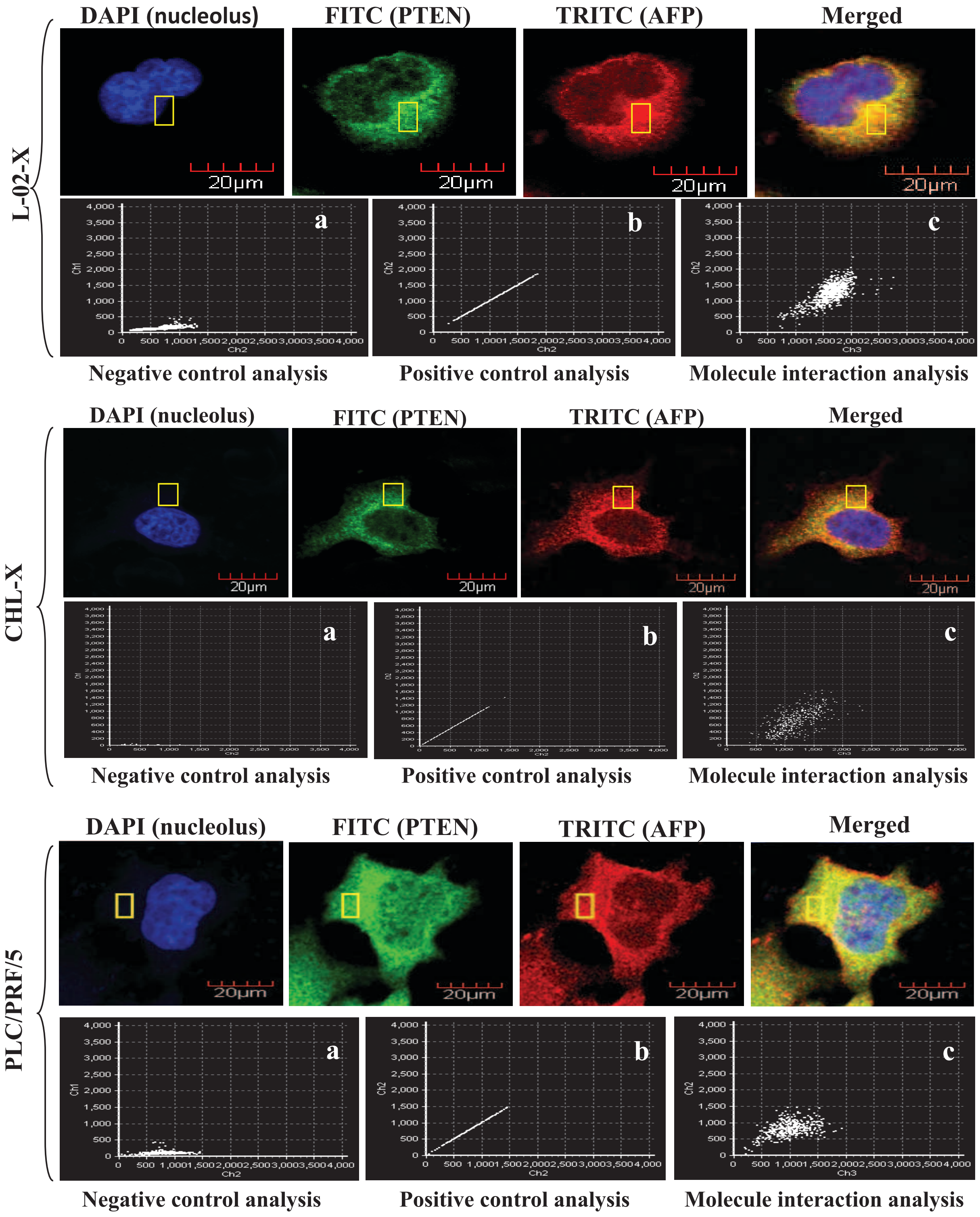

Supplementary Figure S2: The interaction and co-localization of AFP with PTEN in liver cells. Co-localization of AFP and PTEN were assessed in L-02-X, CHL-X, and PLC/PRF/5 cells by laser confocal microscopy. The yellow rectangle denotes the area of the image selected for analysis. Nuclei were stained with DAPI (blue), and PTEN and AFP were labeled with FITC (green) and TRITC (red), respectively. The yellow rectangle denotes the area of the image selected for analysis. The distance between donor (FITC-labeled PTEN) and acceptor (TRITC-labeled AFP) was reflected by the image color.

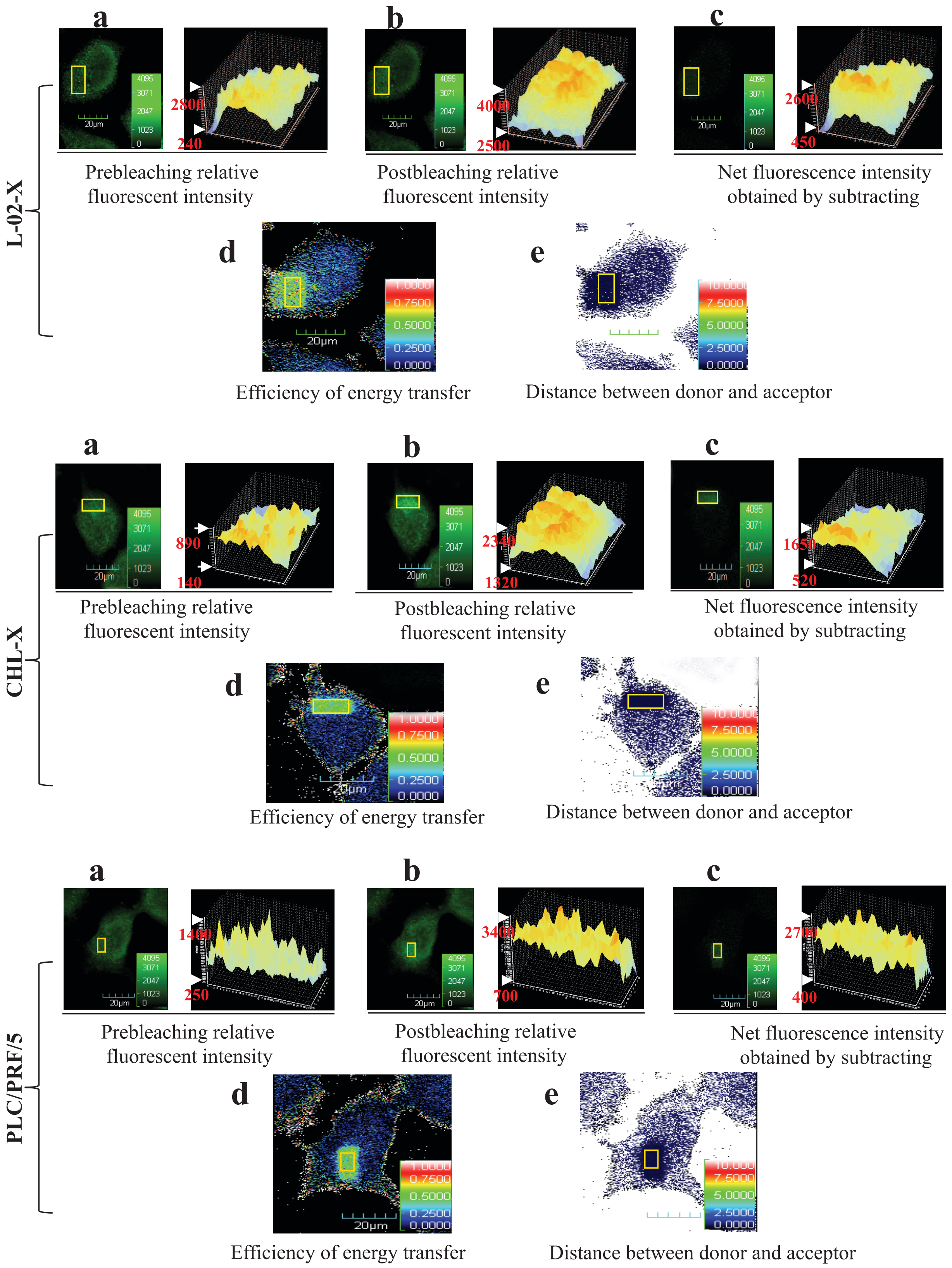

Supplementary Figure S3: The interaction and co-localization of AFP with PTEN in liver cells. FRET analysis also measured AFP and PTEN co-localization. The transition from red to black indicated a gradually decreasing distance between donor and acceptor. Results are from one representative experiment of three.

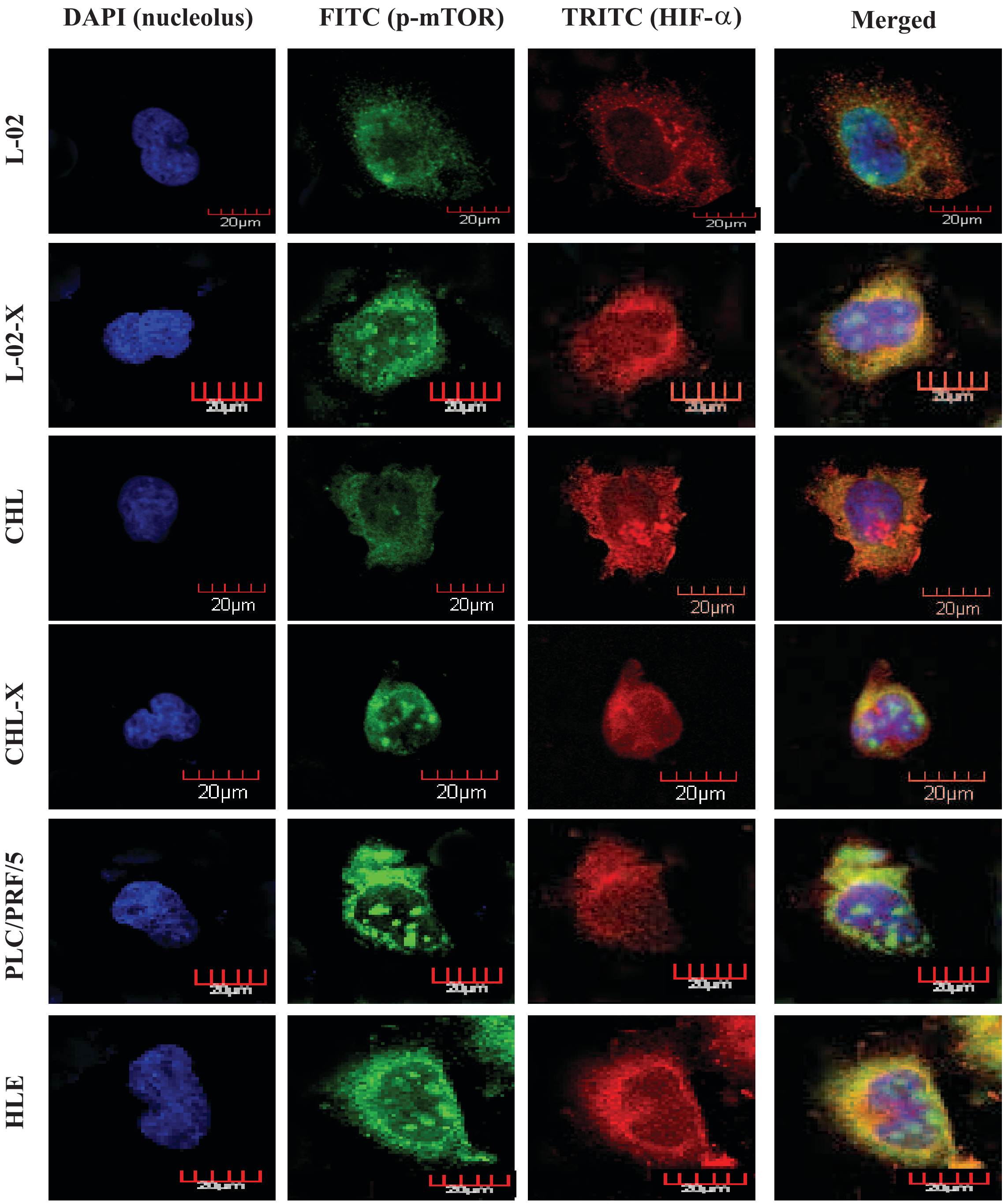

Supplementary Figure S4: Co-localizations of p-mTOR(Ser2448) and HIF-1α in L-02, CHL, PLC/PRF/5, HLE, L-02-X, and CHL-X cells were observed by laser confocal microscopy.

Supplementary Table 1: Patient information

Patient (No.)	Sex[a]	Age	HBsAg[b]	Diagnosis	AFP (ng/ml)
1	M	36	−	Liver trauma	N[c]
2	F	59	−	Liver trauma	N
3	M	53	−	Liver trauma	N
4	F	42	−	Liver trauma	N
5	M	45	−	Liver trauma	N
6	M	58	−	Liver trauma	N
7	M	55	+	Hepatobiliary-stone	N
8	F	53	+	Hepatobiliary-stone	N
9	F	37	+	Hepatobiliary-stone	N
10	M	49	+	Hepatobiliary-stone	N
11	M	48	+	Hepatobiliary-stone	N
12	F	55	+	Hepatobiliary-stone	N
13	M	46	+	Hepatobiliary-stone	N
14	M	37	+	Hepatobiliary-stone	N
15	F	43	−	Hepatobiliary-stone	N
16	M	22	−	Hepatobiliary-stone	N
17	F	65	−	Hepatobiliary-stone	N
18	M	46	−	Hepatobiliary-stone	N
19	M	63	−	Hepatobiliary-stone	N
20	M	58	−	Hepatobiliary-stone	N
21	M	57	−	Hepatobiliary-stone	N
22	F	46	+	Cirrhosis	322
23	M	57	+	Cirrhosis	74
24	M	44	+	Cirrhosis	98
25	F	50	+	Cirrhosis	89
26	M	48	+	Cirrhosis	72
27	M	45	+	Cirrhosis	93
28	M	61	+	Cirrhosis	122
29	M	64	+	Cirrhosis	492
30	F	52	+	Cirrhosis	85
31	M	48	+	Cirrhosis	62
32	M	62	+	Cirrhosis	97
33	F	41	+	Cirrhosis	189
34	M	44	+	Cirrhosis	276
35	F	46	−	Cirrhosis	103
36	F	50	−	Cirrhosis	109
37	M	39	−	Cirrhosis	N

(*Continued*)

Patient (No.)	Sex[a]	Age	HBsAg[b]	Diagnosis	AFP (ng/ml)
38	F	57	–	Cirrhosis	N
39	M	62	+	Cirrhosis	254
40	M	43	+	Cirrhosis	298
41	M	56	+	HCC	269
42	M	61	+	HCC	483
43	M	34	+	HCC	525
44	F	60	+	HCC	116
45	M	38	+	HCC	198
46	M	46	+	HCC	388
47	F	76	+	HCC	445
48	F	32	+	HCC	576
49	M	42	+	HCC	596
50	M	43	+	HCC	295
51	M	57	+	HCC	312
52	F	59	+	HCC	289
53	M	58	+	HCC	277
54	M	52	+	HCC	528
55	M	40	+	HCC	1546
56	M	48	+	HCC	273
57	M	52	–	HCC	N
58	F	63	–	HCC	N
59	F	39	–	HCC	1324
60	M	42	+	HCC	678
61	M	36	–	HCC	244
62	F	44	–	HCC	N
63	M	41	+	HCC	536

[a], "M", male; "F", female
[b], The liver tissue contained HBV were confirm by patients' serum HBsAg; "+", positive; "–", negative
[c], "N", negative, patients' serum concentration of AFP < 40 ng/ml.
